# Integrase inhibitor-based treatment in clinical practice

**DOI:** 10.1186/1758-2652-13-S4-P32

**Published:** 2010-11-08

**Authors:** G Cenderello, R Piscopo, M Feasi, G Penco, N Bobbio, G Cassola

**Affiliations:** 1 EO Ospedali Galliera, Infectious Diseases, Genoa, Italy

## Background

Raltegravir(RAL) is a the first compound of a novel class: integrase inhibitors. Aim of our study was to evaluate efficacy and safety of RAL based regimen.

## Materials and methods

We proceeded to retrospective analysis from patients(pts) referring to our service. We searched our databases for epidemiological, clinical, immunological and virological issues. Moreover we recorded HCV and HBV status. We focused our attention on the reason of ARV switching , duration of ARV before RAL introduction, immunological gain, virological suppression and tolerability of the prescribed regimen. We followed side effects in order to define safety of prescribed regimen.

## Results

We enrolled 70 pts. (male 53:female 17), according to CDC 1993 they were classified as A(12); B(32);C(26). Median age was 49 years, 42 pts were HCV-positive and only 4 presented an active HBV coinfection. Risk factors for HIV infection resulted: injective drug abuse(33) homosexual(20) and heterosexual(17)exposition. They were switched to RAL therapy for drug resistance(39)(DRR); regimen simplification(7) or drug toxicity (24). Most of patients presented multiple drug failures to ARV therapy (almost two different classes resistance). Median follow up was with RAL is 9 months and median extent of ARV before RAL was 9 years. Among DRR. 29 were coming from a PI failure whereas 9 from NNRTI based regimen and one from 3TC monotherapy. The most represented drug associated in new ARV regimen was DRV/rtv (42) followed by etravirine(12) and maraviroc (10).

We collected data of immune and virological response after 3 and 6 months, so at 6 months 59 pts. (84.3%; 71.8 % in DRR) presented undetectable(<20) viral load and a medium CD4 gain of 89 cc/ml(90 cc/ml in DRR). No relevant side effects were observed.

## Conclusions

Pts. treated with RAL based therapy presented a good outcome, most of them achieve (after 6 months) virological suppression and immunological recovery. In our case list, darunavir/rtv and RAL obtained the best results with no relevant side effects. So more studies are needed to confirm that RAL based ARV could be very efficacious and safe even in long term follow up period.

